# Alemtuzumab in Multiple Sclerosis: Mechanism of Action and Beyond

**DOI:** 10.3390/ijms160716414

**Published:** 2015-07-20

**Authors:** Tobias Ruck, Stefan Bittner, Heinz Wiendl, Sven G. Meuth

**Affiliations:** Department of Neurology, University of Muenster, Muenster 48149, Germany; E-Mails: stefan.bittner@ukmuenster.de (S.B.); heinz.wiendl@ukmuenster.de (H.W.); sven.meuth@ukmuenster.de (S.G.M.)

**Keywords:** alemtuzumab, CD52, mechanism of action, secondary autoimmune disease, multiple sclerosis, experimental autoimmune encephalomyelitis (EAE)

## Abstract

Alemtuzumab is a humanized monoclonal antibody against CD52 (cluster of differentiation 52) and is approved for the therapy of relapsing-remitting multiple sclerosis. The application of alemtuzumab leads to a rapid, but long-lasting depletion predominantly of CD52-bearing B and T cells with reprogramming effects on immune cell composition resulting in the restoration of tolerogenic networks. Alemtuzumab has proven high efficacy in clinical phase II and III trials, where interferon β-1a was used as active comparator. However, alemtuzumab is associated with frequent and considerable risks. Most importantly secondary autoimmune disease affects 30%–40% of patients, predominantly impairing thyroid function. Extensive monitoring and early intervention allow for an appropriate risk management. However, new and reliable biomarkers for individual risk stratification and treatment response to improve patient selection and therapy guidance are a significant unmet need. Only a deeper understanding of the underlying mechanisms of action (MOA) will reveal such markers, maximizing the best potential risk-benefit ratio for the individual patient. This review provides and analyses the current knowledge on the MOA of alemtuzumab. Most recent data on efficacy and safety of alemtuzumab are presented and future research opportunities are discussed.

## 1. Introduction

Multiple sclerosis (MS) is a chronic inflammatory disorder of the central nervous system (CNS) characterized by inflammation, demyelination, and axonal degeneration. Around 2.5 million people suffer from MS worldwide. In the recent years new drugs have revolutionized MS therapy. However, there is often only limited knowledge on the mechanisms of action underlying the effects and adverse events of these therapeutic agents, what might be especially true for alemtuzumab. Alemtuzumab is a humanized monoclonal antibody targeting CD52, a surface molecule with largely unknown function and which is predominantly expressed on B and T cells [1]. Since the approval of the U.S. Food and Drug Administration (FDA) in November 2014, alemtuzumab is approved in over 30 countries for the therapy of relapsing-remitting multiple sclerosis (RRMS). Alemtuzumab demonstrated superiority to subcutaneous (s.c.) interferon β-1a (IFNβ-1a), an established disease-modifying therapy (DMT) for RRMS. The high efficacy of alemtuzumab, with remarkable effects on clinical and radiological disease outcome parameters is offset by frequent and significant adverse events. Besides infusion-associated reactions (IAR), mild to moderate infections, especially the occurrence of secondary autoimmune disease, has somewhat dampened enthusiasm for alemtuzumab. Approximately 30%–40% of patients develop secondary autoimmune diseases predominantly affecting thyroid, kidney and thrombocytic function [2]. However, the implementation of a safety monitoring program allows for the early detection and management of these autoimmune events.

Alemtuzumab leads to a rapid and long-lasting depletion of CD52 positive cells, followed by a slow repopulation arising from unaffected hematopoietic precursor cells. Besides quantitative changes of the immune cell repertoire, also qualitative alterations of immune components are seen enabling a rebalancing of immune-tolerance networks. The exact mechanisms underlying reprogramming of the immune system are far from being understood [3].

This review focuses on the mechanisms of action underlying clinical efficacy and the adverse event profile of alemtuzumab. Data from recent animal and *ex vivo* human studies as well as from clinical trials are analyzed. Moreover, the newest data on efficacy and safety of alemtuzumab are summarized, and an outlook on future research opportunities is given. 

## 2. Alemtuzumab—Efficacy, Safety, Mechanisms and Perspectives

### 2.1. Alemtuzumab—A Humanized Anti-CD52 Antibody

Alemtuzumab (Campath-1H) is a monoclonal IgG1 antibody binding to the human CD52 protein. It is a humanized antibody with a rat-derived antigen-specific, highly variable Fab region and an Fc region of human origin [4,5]. CD52 is a glycosylphosphatidylinositol (GPI)-anchored protein consisting of 12 amino acids expressed at high levels on T and B lymphocytes, and to a lesser extent on monocytes, macrophages and eosinophil granulocytes. Mature natural killer (NK) cells, plasma cells, neutrophil granulocytes, and most importantly, hematological stem cells show little or no expression [6,7,8]. However the exact physiologic function of CD52 is still largely unknown.

Although not possessing intracellular domains, CD52 has been implicated in the activation and migration of T lymphocytes [9,10,11]. Of note, Watanabe and colleagues demonstrated CD52 as a co-stimulatory molecule inducing regulatory CD4^+^CD25^high^ T cells [12]. The monoclonal antibody 4C8 as well as alemtuzumab bound to CD52. When immobilized to cell culture plates with suboptimal doses of anti-CD3 these antibodies led to the generation of regulatory T cells (anti-CD52 induced Tregs) providing effective, cell-contact dependent and cytokine-independent suppression of *in vitro* and *in vivo* T cell responses. The forkhead transcription factor FoxP3, regarded as the master regulator in development and function of thymus-derived regulatory T cells, was only transiently expressed at low levels arguing for a distinct and independent subset of Tregs. Whether these cells are generated *in vivo* in alemtuzumab-treated MS patients, and their functional contribution to the MOA of alemtuzumab has not been investigated so far. Moreover, it is unclear if these cells are related to the recently identified CD52^high^CD4^+^ regulatory T cells [13]. In contrast to anti-CD52 induced Tregs, the suppressive function of CD52^high^CD4^+^ regulatory T cells is cell-contact independent and mediated by soluble CD52 released by phospholipase C. Siglec-10 was identified as a possible receptor to soluble CD52 impairing phosphorylation of the T cell receptor-associated kinases Lck and Zap70, and thus T cell activation. Patients with type 1 diabetes showed lower frequencies and diminished function of CD52^high^CD4^+^ T cells responsive to the autoantigen GAD65, but not to tetanus toxoid suggesting an antigen-specific reduction of suppressive activity. Alterations in frequency and function of thymus-derived FoxP3^+^ Tregs have been implicated in the pathogenesis of MS [14,15], whether CD52^high^CD4^+^ T cells are involved in MS pathology has not been investigated so far. The high CD52 expression levels render this cell type very susceptible to alemtuzumab-mediated cell lysis. Nevertheless alemtuzumab effectively suppresses neuroinflammatory responses in MS. Therefore, CD52^high^CD4^+^ T cells presumably do not play a crucial role in the inflammatory processes of MS. However, their depletion might be of importance in the development of secondary autoimmune disease after alemtuzumab treatment. CD52^high^CD4^+^ T cells might be preferentially activated by antigens related to the thyroid gland, the kidneys or thrombocytes possibly explaining the high prevalence of autoimmunity directed to these organs after alemtuzumab application. A better understanding of the cellular functions of CD52 will be of great value to clarify the effects of alemtuzumab.

According to the labeling information, 12 mg of alemtuzumab are infused for five consecutive days in the first course and for three days in the second course one year later. Currently alemtuzumab therapy is approved for the initial two infusions; however there are reports of patients having already received up to five courses [16]. Concomitant corticosteroids, antihistamine and antipyretic drugs are applied with the infusion in order to avoid infusion-related reactions (IAR).

Although there is only sparse knowledge on physiological CD52 function, the immediate effects of alemtuzumab on CD52 expressing cells are quite clear. Within a few minutes after infusion alemtuzumab leads to depletion of CD52 positive cells through antibody-dependent cell-mediated cytolysis (ADCC) and complement-dependent cytolysis (CDC). Human lymphocytes are susceptible to ADCC and CDC [17], however in the mouse model depletion is predominated by neutrophil and NK cell mediated ADCC [18]. Although the drug’s half-life accounts only for four to five days in MS patients [19], after depletion a very slow repopulation starts arising from hematopoietic precursor cells following a distinct temporal pattern. Monocytes reach baseline levels after three months. B cell counts not only return to baseline numbers by three months, but they also show an increase to 124%–165% of baseline levels at 12 months [20,21]. CD8^+^ T lymphocytes are restored after 31 months, while CD4^+^ T lymphocytes need around 60 months for complete repopulation [20]. Sustained high levels of apoptosis may explain the prolonged T cell lymphopenia in MS despite not affecting hematologic precursors [22].

### 2.2. Clinical Efficacy of Alemtuzumab: Phase II/III Trials and Newest Data

Since 1991, MS patients at Addenbrooke’s Hospital in the United Kingdom (UK) have been treated with alemtuzumab. These early investigations revealed alemtuzumab’s high efficiency in ameliorating inflammatory responses in relapsing-remitting as well as in secondary progressive MS (SPMS). Despite a strong reduction in relapse-rate and MRI disease activity, alemtuzumab was not able to prevent disability progression in SPMS patients [20,23]. Accordingly, the focus of alemtuzumab research was shifted to RRMS patients in consecutive trials.

The Phase II trial CAMMS223 was a randomized, rater-blinded study enrolling 334 patients (216 patients received alemtuzumab). Alemtuzumab was tested against an active comparator with s.c. IFNβ-1a. The co-primary endpoints were annualized relapse rate (ARR) and six-month sustained accumulation of disability (SAD; defined as sustained increase for at least six months in EDSS (Expanded Disability Status Scale), of ≥1.5 EDSS points if baseline EDSS was 0; ≥1.0 points if the baseline EDSS was ≥1) [24]. Co-primary endpoints were met and have been reviewed in detail together with study design elsewhere [2,19]. Briefly, compared with s.c. IFNβ-1a, ARR was reduced by 74% (0.1 *vs.* 0.36; *p* < 0.001) and SAD by 71% (9% *vs.* 26.2%; *p* < 0.001). Notably, the alemtuzumab group demonstrated an improvement of 0.39 EDSS points, while the mean EDSS of the s.c. IFNβ-1a group deteriorated by 0.38 points. In a *post hoc* analysis at three years, alemtuzumab led to lack of clinical disease activity (defined as absence of relapses and six-month SAD) in 72% *vs.* 43% of s.c. IFNβ-1a treated patients (HR = 0.33; *p* < 0.0001) [25]. Another newly introduced outcome parameter, sustained reduction in disability (SRD, defined as EDSS reduction of ≥1.0 points sustained for at least six months in patients with baseline EDSS ≥ 2.0) also demonstrated the superiority of alemtuzumab over s.c. IFNβ-1a (45% *vs.* 27% at year three; *p* = 0.01).

The two Phase III trials, CARE-MS (Comparison of Alemtuzumab and Rebif^®^ Efficacy in Multiple Sclerosis) I and II, were randomized, rater-blinded studies with s.c. IFNβ-1a as active comparator designed to test clinical application of alemtuzumab. CARE-MS I included 581 treatment-naïve RRMS patients (EDSS score ≤ 3.0, MS symptom onset within five years), whereas CARE-MS II enrolled 637 RRMS patients with break-through disease under previous DMTs (EDSS score ≤ 5.0, MS symptom onset within 10 years) [26,27]. The study design and efficacy parameters have been reviewed in detail elsewhere [2,19,28]. Most of the CAMMS223 (A Phase II Study Comparing Low- and High-Dose Alemtuzumab and High-Dose Rebif^®^ in Patients With Early, Active Relapsing-Remitting Multiple Sclerosis) results were confirmed. Alemtuzumab demonstrated a superior reduction in ARR compared to s.c. IFNβ-1a therapy (0.18 *vs.* 0.39 = 54.9% ARR reduction in CARE-MS I; 0.26 *vs.* 0.52 = 49.4% ARR reduction in CARE-MS II; each with *p* < 0.0001). However, a significant reduction in six-month SAD was only observed in CARE-MS II (42% reduction; 21% with s.c. IFNβ-1a *vs.* 13% with alemtuzumab; *p* < 0.01), but not in CARE-MS I (11% with s.c. IFNβ-1a *vs.* 8% with alemtuzumab; *p* = 0.22). The latter might be attributed to the unexpectedly low rate of disability progression in the s.c. IFNβ-1a group, indicating a relatively underpowered trial. MRI measures also proved superiority of alemtuzumab with significantly less gadolinium-enhancing lesions, new or enlarging T2 lesions and brain atrophy. Significantly more alemtuzumab than s.c. IFNβ-1a treated patients were free of any clinical disease (CARE-MS I: 74% *vs.* 56%, *p* < 0.0001; CARE-MS II: 60% *vs.* 41%; *p* < 0.0001) and free of any clinical and MRI disease activity (CARE-MS I: 39% *vs.* 27%, *p* < 0.01; CARE-MS II: 32% *vs.* 14%; *p* < 0.0001). 

Patients completing Phase II and III trials were eligible to continue in ongoing extension studies. In a five-year follow-up of the CAMMS223 extension study (now included in CARE-MS extension) alemtuzumab demonstrated durable effects on relapse rate (ARR reduced by 69%, *p* < 0.0001; 9/151 patients needed additional alemtuzumab treatment due to breakthrough disease), disability (SAD reduced by 72%, *p* < 0.0001; EDSS remained stable or improved in 74% of the patients) and MRI measures [29]. Preliminary data on the CARE-MS extension studies (CARE-MS I: 349 patients, CARE-MS II: 393 patients) have been presented at the ECTRIMS (European Committee for Treatment and Research in Multiple Sclerosis) Congress 2014 and AAN (American Academy of Neurology) Congress 2015. The four-year follow-up showed alemtuzumab retreatment rates between 36% for CARE-MS I and 32% for CARE-MS II patients over three years since the initial two treatment courses. Only 2%–5% of patients received other DMTs in years three and four. No evidence of disease activity (NEDA; defined as absence of clinical disease and MRI activity) was observed in 50%–60% of patients in years three and four [30,31]. CARE-MS II patients demonstrated a mean EDSS score of 2.7 by year four, which did not increase compared to the core study baseline, 38.6% of the patients showed improved and 27.6% stable EDSS scores. Of 330 patients, six-month SRD was achieved in 41%, and 12-month SRD in 30%, over four years [32]. As seen in the five-year follow-up of the CAMMS-223 extension study, alemtuzumab also had durable effects on MRI outcome measures. The slowing of brain volume loss measures exemplified by the median yearly brain parenchymal fraction (BPF) change was maintained over four years (CARE-MS I: end of core study = −0.25%, year three = −0.19%, year four = −0.15%; CARE-MS II: end of core study = −0.22%, year three = −0.10%, year four = −0.19%) [33]. Around 70% of patients demonstrated freedom of MRI disease activity at years three and four (CARE-MS I: 72.1%/70.1%; CARE-MS II: 68.4%/69.9%) [34,35]. Switching from s.c. IFNβ-1a to alemtuzumab improved several clinical and MRI parameters (ARR, freedom of relapses [36,37] and MRI disease activity [38,39], BPF loss [40] yielding comparable outcomes at year two as have been observed for alemtuzumab-treated patients at the end of the core study. However, switching from s.c. IFNβ-1a did not considerably change the safety profile of alemtuzumab.

The most important efficacy outcome measures of the Phase II and III trials are summarized in Table 1.

**Table 1:** Efficacy endpoints of Phase II and III clinical trials on alemtuzumab in RRMS.

Parameter	CAMMS223 ^a^ [24,25]	CAMMS223 Extension ^a^ [29]	CARE-MS I [26]	CARE-MS II ^a^ [27]
Number of patients	A: *n* = 112, IFN: *n* = 111	A: *n* = 112, IFN: *n* = 111	A: *n* = 376, IFN: *n* = 187	A: *n* = 202, IFN: *n* = 426
ARR at study end (reduction by alemtuzumab)	A: 0.11, IFN: 0.36 (69%, *p* < 0.001)	A: 0.12, IFN: 0.35 (66%, *p* < 0.0001)	A: 0.18, IFN: 0.39 (55%, *p* < 0.0001)	A: 0.26, IFN: 0.52 (49%, *p* < 0.0001)
SAD (reduction by alemtuzumab)	A: 8%, IFN: 24% (75%, *p* < 0.001)	A: 13%, IFN: 30% (69%, *p* < 0.001)	A: 8%, IFN: 11% (*p* = 0.22)	A: 13%, IFN: 21% (42%, *p* < 0.01)
Mean EDSS change	A: −0.32, IFN: +0.46 (*p* < 0.001)	A: −0.15, IFN: +0.46 (*p* = 0.014)	A: −0.14, IFN: −0.14 (ns)	A: −0.17, IFN: +0.24 (*p* < 0.0001)
SRD	A: 45%, IFN: 27% (year 3, *p* = 0.01)	n/a	A: 23%, IFN: 25% (ns)	A: 29%, IFN: 13% (*p* = 0.0002)
Relapse-free patients	A: 77%, IFN: 52% (*p* < 0.001)	A: 68%, IFN: 41%	A: 78%, IFN: 59% (*p* < 0.0001)	A: 65%, IFN: 47% (*p* < 0.0001)
Freedom of clinical disease	A: 72%, IFN: 43% (year 3, *p* < 0.001)	n/a	A: 74%, IFN: 56% (*p* < 0.0001)	A: 60%, IFN: 41% (*p* < 0.0001)
Gd-enhancing lesions	n/a	n/a	A: 15%, IFN: 27% (*p* = 0.001)	A: 19%, IFN: 34% (*p* < 0.0001)
New/enlarging T2 lesions	n/a	n/a	A: 49%, IFN: 58% (*p* = 0.04)	A: 46%, IFN: 68% (*p* < 0.0001)
Median change in T2 lesion volume	A: −18.2%, IFN: −13.3% (ns)	n/a	A: −6.5%, IFN: −9.3% (ns)	A: −1.2%, IFN: −1.3% (ns)
Freedom of MRI and clinical disease	n/a	n/a	A: 39%, IFN: 27% (*p* = 0.006)	A: 32%, IFN: 14% (*p* < 0.0001)
Median change in BPF from baseline	A: −0.9%, IFN: −1.8% (ns)	n/a	A: −0.9%, IFN: −1.5% (*p* < 0.0001)	A: −0.6%, IFN: −0.8% (*p* = 0.012)

^**a**^: Only patients in the 12 mg alemtuzumab treatment arm are displayed for better comparability; A: alemtuzumab; ARR: annualized relapse rate; BPF: brain parenchymal fraction; EDSS: Expanded Disability Status Scale; Gd: gadolinium; IFN: s.c. interferon β-1a; n/a: not available; ns: not significant; RRMS: relapsing-remitting multiple sclerosis; SAD: six-month sustained accumulation of disability; SRD: six-month sustained reduction of disability.

### 2.3. Safety Profile of Alemtuzumab

The high efficacy of alemtuzumab contrasts with its considerable risks. Safety data are gained from the early Cambridge cohort (12-year data), CAMMS223 extension (seven-year data) and CARE-MS extension (four-year data) trials. So far the extension studies provide no new safety signals compared to the key clinical trials, however only long-term data will warrant definite conclusions. Of note, the s.c. IFNβ-1a groups showed higher study discontinuation rates due to AEs throughout the key trials (5.8%–12.1% *vs.* 1.3%–3.0% in alemtuzumab treated patients) [1,24,25,26,29]. Secondary autoimmune disease remains the most important adverse event of alemtuzumab therapy. The experiences of clinical trials led to the implementation of extensive safety measures allowing for early recognition and therapy of adverse events (AE). Table 2 summarizes the most frequent and important adverse events as well as adequate management strategies.

**Table 2:** Adverse events of alemtuzumab, frequency, monitoring and management.

Adverse Event	Prevalence ^a^	Highest Incidence	Risk-Monitoring	Management
IARs	>90%	During infusion and 24 h thereafter	Clinical and technical monitoring of vital signs	Corticosteroids (first 3 days of infusion), antihistamines and/or antipyretics (prior and as needed)
Infections	66%–77%	Year 1	Frequent follow-up visits	Herpes prophylaxis ≥1 month after alemtuzumab
Thyroid disorders	30%–41%	Year 3 Onset 6–61 months	Thyroid function test (e.g., TSH)	Prior to alemtuzumab and quarterly after alemtuzumab for 48 months
ITP	1%–3%	Onset 1–34 months	CBC and differential	Prior to alemtuzumab and monthly after alemtuzumab for 48 months
Glomerulo-nephritis	0.3%	Onset 4–39 months	Serum creatinine and urinalysis with microscopy	Prior to alemtuzumab and monthly after alemtuzumab for 48 months

^**a**^: prevalence over all key clinical trials; CBC: complete blood count; IAR: infusion-associated reaction; ITP: immune thrombocytopenia; TSH: thyroid-stimulating hormone.

#### 2.3.1. Infusion-Associated Reactions (IAR)

IARs were the most common adverse events affecting over 90% of patients [1,24,25,26,29]. They are defined as any AE occurring during infusion or within 24 h after infusion. Most were mild to moderate and consisted of headache, rash, pyrexia and nausea; serious IARs were reported only in 1%–3% across all studies [1]. The rapid release of the cytokines TNF-α, IFN-γ and IL-6 resulting from target cell lysis and consecutive inflammatory responses is assumed to be causal for IARs [41,42]. Corticosteroids, antihistamines and antipyretic drugs are used in pretreatment of alemtuzumab to prevent or ameliorate IARs. The frequency of IARs decreases with repeated treatment courses [43]. A recent study investigating the impact of nonconsecutive alemtuzumab administration (course 1: 6–10 nonconsecutive days, course 2: 4–8 nonconsecutive days) on IARs showed no relevant changes in IAR frequency or severity. Of note, no significant impact on lymphocyte depletion or clinical and MRI outcome parameters was observed [44]. The manufacturer sponsored safety study EMERALD (Evaluation of the Management of Infusion Reactions in Alemtuzumab-Treated Patients) started in September 2014 and aims to assess IARs under the precautions of comprehensive infusion guidance [45].

#### 2.3.2. Infections

Over all key clinical trials infections were more frequent in alemtuzumab treated compared to s.c. IFNβ-1a treated patients (CAMMS223: 65.7% *vs.* 46.7%; CAMMS223 extension: 72.2% *vs.* 50.4%, CARE-MS I 67% *vs.* 45%; CARE-MS II 77% *vs.* 66%). Most infections were classified as mild to moderate, although serious infections occurred more often in the alemtuzumab treatment groups (2.0%–6.9% *vs.* 1.0%–2.8%) [24,26,27,29]. Respiratory tract and urinary tract infections were most common. The accumulations of herpes infections during the CARE-MS studies led to the implementation of prophylactic acyclovir treatment (0–4 weeks after alemtuzumab infusion) significantly reducing infection rates [46]. Moreover, there are single case reports of spirochetal gingivitis, pyogenic granuloma, esophageal candidiasis, tuberculosis and listeria meningitis; the latter leading to dietary advice to avoid, for example, unpasteurized cheese [1,2]. No cases of progressive multifocal leukoencephalopathy (PML) have been reported so far.

Follow-up data from the CAMMS223 extension demonstrated decreasing incidences with each course of alemtuzumab [29] corroborated by recent data from four-year follow-up of the CARE-MS extension [47]. The infection incidence was highest in year one after alemtuzumab treatment, and declined thereafter (year one: 59.9%, year two: 55.1%, year three: 48.2%, year four: 46%). As described before, infections were mostly mild or moderate in severity with none leading to withdrawal of treatment or study discontinuation.

Whether vaccinations or preventive medications other than acyclovir are able to reduce infection incidence has not been investigated so far.

However, given the profound and long-term lymphopenia after alemtuzumab, the frequency of opportunistic or severe infections is still unexpectedly low. The rare occurrence might be attributed to the relative preservation of innate immune responses and, compared to the blood, less pronounced lymphocyte depletion in primary and secondary lymphoid tissues [18]. Innate immune cells express only low or no CD52 rendering them more or less resistant to alemtuzumab-mediated depletion. Histological stainings indicated that human innate immune cells might even lose more CD52 during maturation or differentiation [48]. In humanized CD52 mice, lymphocyte depletion after alemtuzumab was significantly less in spleen, bone marrow, lymph nodes and thymus than in blood [18]. Accordingly, alemtuzumab-treated patients are still able to exert protective B and T cell responses following vaccination [49]. Moreover, skin-resident effector memory T cells were spared when alemtuzumab was administered to patients with cutaneous T cell lymphomas [50].

#### 2.3.3. Malignancy

As stated above the lack of long-term data does not allow for definite conclusions concerning alemtuzumab’s risks, especially in case of malignancies. However, 29 patients so far have been diagnosed with a malignancy in the clinical development program of alemtuzumab [51]. Of note, six of these cases presented with thyroid carcinomas, most of them classified as papillary thyroid cancers. Whether this increased incidence of thyroid malignancies is attributable to the intensive surveillance of thyroid function or to alemtuzumab treatment itself has to be clarified. Additionally, six cases of basal cell carcinoma, five cases of breast cancer and four cases of malignant melanoma have been observed among other malignancies.

#### 2.3.4. Pregnancy

According to the labeling information, women of childbearing potential are recommended to use effective contraception during and four months after alemtuzumab infusion. Breastfeeding should be stopped during treatment and for four months afterwards. Alemtuzumab has been assigned to pregnancy category C by the FDA, since there are no controlled data in human pregnancies and IgG antibodies are able to cross the placenta. In animal studies with pregnant humanized CD52 transgenic mice, alemtuzumab showed no teratogenic effects when administered during organogenesis; however, there was an increase in embryo lethality [52]. Moreover, autoantibodies developing after alemtuzumab treatment might be transferred via the placenta and harm the unborn child. As of October 2013, under alemtuzumab treatment 139 pregnancies in 104 patients were reported resulting in 67 live births, 38 abortions (14 elective, 24 spontaneous), one stillbirth, and 33 with unknown outcome. In 11 pregnancies serious AEs were reported (two fetuses with cystic hygroma and hypoplastic heart, one intrauterine death due to nuchal cord) [53]. Male fertility seems to be unaffected; however large scale studies are missing [54].

#### 2.3.5. Secondary Autoimmune Disease

Autoimmunity is the most important and substantial adverse event (AE) associated with alemtuzumab treatment. Over all key clinical trials including extension studies, thyroid autoimmune disease, occurring in 30%–41% of patients, was most common. The onset ranged from six to 61 months peaking in year three and declining thereafter [16,55]. Thyroid autoimmune disease mainly comprised hyperthyroidism, hypothyroidism, goiter and thyroiditis. One to three percent developed immune thrombocytopenia (ITP), with the index patient presenting with a lethal intracranial hemorrhage. The onset ranged from one to 34 months post alemtuzumab. Additionally, four cases of glomerulonephritis (0.3% of patients, two with Goodpasture syndrome, two with membranous glomerulonephritis; onset four to 39 months), and single cases of autoimmune neutropenia, hemolytic anemia and type 1 diabetes have been reported [1,28]. Autoimmune phenomena such as asymptomatic autoantibodies occur in one-third of patients [56]. Secondary autoimmune disease—at least in the case of thyroid AEs—seems to have no impact on the efficacy of alemtuzumab [57].

Analog autoimmune responses have been reported in other lymphopenic conditions (e.g., after bone marrow transplant and HIV therapy), however not all lymphopenic individuals develop autoimmunity, therefrom a “two-hit model” of lymphopenia associated autoimmunity has been proposed [58]. Lymphopenia, the first hit, promotes the proliferation of self-antigen responsive T cells, which in the case of alemtuzumab have escaped depletion. This process is termed homeostatic proliferation and is driven by recognition of self-MHC/peptide ligands and cytokines such as IL-21 [59,60]. IL-21, as possible “second hit”, was shown to propagate T cell cycling increasing the stochastic probability of self-antigen encounter [22]. Moreover, IL-21 has been implicated in the induction of Th17 cells [61], in B cell differentiation and antibody production [62], and inhibition of Treg function [63,64]. Increased serum IL-21 concentrations related to a specific genetic risk profile have been shown to correlate with the incidence of autoimmune disease after alemtuzumab [22]. Another recent study from Jones and colleagues further argues for the concept of homeostatic proliferation as an important factor in alemtuzumab-associated autoimmunity [56]. T cell recovery in the first 6–12 months after alemtuzumab was mainly driven by homeostatic proliferation as the T cell pool was dominated by chronically activated, highly proliferative, oligoclonal, effector memory T cells. Patients developing autoimmune disease demonstrated reduced thymopoiesis 12 months after alemtuzumab and exhibited clonal restriction of the T cell repertoire. No differences in CD4^+^ and CD8^+^ T cell reconstitution were observed. However, these alterations were pronounced in the early phase after alemtuzumab, how this relates to autoimmunity peaking in year two to three remains to be clarified.

Besides elevated IL-21, reduced IL-7 levels correlated with an increased risk of autoimmune disease after alemtuzumab. IL-7 stimulates thymic output, therefore decreased levels might contribute to the imbalance of homeostatic proliferation and thymopoiesis [65,66].

Further potential “second hits” might be genetic and/or environmental factors. The occurance of autoimmune thyroid disorders, nephropathies and cytopenia in MS patients after alemtuzumab treatment is often explained by genetic risk profiles. Indeed, there is an increased incidence of Graves’ disease in family members of MS patients [67]. Goodpasture syndrome after alemtuzumab was shown to be associated with the susceptibility allele HLA-DRB1-15 [68], which is also a strongly linked genetic risk factor for MS [69]. A genetic association between immune thrombocytopenia and MS has also been suggested [70]. However, there also have been reports for the coexistence of MS and diabetes, rheumatoid arthritis, systemic lupus erythematosus and myasthenia gravis weakening the line of argumentation [71]. Age and sex seem to have no impact on alemtuzumab-associated autoimmune disease, whereas smoking was found as a relevant risk factor (odds ratio: 3.05, 95% CI 1.50–6.19) [70].

The most frequent autoimmune AEs after alemtuzumab are predominantly B cell driven and auto-antibody mediated, providing an alternative explanation; the faster B cell than T cell recovery after alemtuzumab might set the stage for unregulated B cell expansion and antibody-production in response to self-antigens [3]. Overshooting B cell recovery coincides with increased serum BAFF (B cell activating factor) concentrations, which have previously been associated with B cell-related autoimmune disorders [21].

Frequencies of CD4^+^CD25^high^CD127^low^ Tregs are relatively increased to other CD4^+^ T cell subsets for up to nine months after alemtuzumab [72,73]. Reliable data on their regulatory capacity early after alemtuzumab is still missing due to very low recoverable cell numbers. However, recently it was reported that patients three to four years after treatment show restored suppressive Treg function equivalent to healthy controls. Interestingly, no difference in suppressive Treg function was found in patients with or without autoimmune disease [56], although, low Treg numbers or still compromised Treg function might have been permissive for the development of autoimmunity before this time point. As stated above, the depletion of autoantigen-specific regulatory T cell populations such as CD52^high^CD4^+^ T cells might also contribute to secondary autoimmune responses.

Table 3 summarizes the possible mechanisms underlying secondary autoimmune disease following alemtuzumab administration in MS patients.

**Table 3:** Lymphopenia-associated secondary autoimmune disease after alemtuzumab.

Potential Mechanism	Consequences	References
Homeostatic proliferation ↑	Chronically activated, oligoclonal, auto-reactive T cells ↑	[56,59,60]
IL-21 ↑	Homestatic proliferation ↑	[22]
	Th17 cells ↑	[61]
	B cell differentiation, antibody production	[62]
	Treg function ↓	[63,64]
Thymopoiesis ↓	Clonal restricted T cell receptor repertoire	[56]
IL-7 ↓	Thymic output ↓, homeostatic proliferation > thymopoiesis	[65,66]
Faster B cell recovery than T cell recovery, BAFF ↑	Unregulated B cell expansion in response to self-antigens	[3,74]
Low absolute Treg numbers, potentially compromised Treg function ^a^	Diminished control of autoimmune responses	[13,72,73]
Genetic risk profile	Susceptibility to autoimmunity ↑	[67,69,70]
Smoking	Susceptibility to autoimmunity ↑	[70]

^a^: including thymus-derived CD4^+^CD25^high^FoxP3^+^ and other CD52-bearing regulatory T cells, such as CD52^high^CD4^+^ T cells; BAFF: B cell activating factor; Th: T helper cell; Treg: regulatory T cell.

### 2.4. Mechanisms of Action (MOA)

The clinical effects of alemtuzumab have been extensively studied. Despite growing knowledge the underlying MOAs remain largely unknown. Table 4 summarizes the current knowledge on alemtuzumab’s MOA.

**Table 4:** Mechanisms of action of alemtuzumab.

Effect	Potential Mechanism	References
Infusion-associated reactions (e.g., fever, rash, malaise)	TNF-α, IFN-γ and IL-6 release by target cell lysis and consecutive inflammatory responses	[41,42]
Reduction of MS-related inflammatory responses	Depletion of CD52^+^ circulating immune cells	[18,21,72]
	Prolonged T cell lymphopenia (CD4 > CD8)	[22,72]
	Prolonged memory B cell lymphopenia	[21]
	Maturation of immune cells in a tolerogenic environment: relative Treg numbers ↑, restored Treg function, TGF-β ↑, IL-10 ↑, IFN-γ ↓, IL-12 ↓, IL-17 ↓, Th1 ↓, Th2 ↑, Th17 ↓, expression of inhibitory receptors on T cells: PD-1 ↑, LAG-3 ↑	[56,75]
	Reduction of autoreactive T cell clones, increased TCR diversity	[56,76,77]
	Reduced T cell migration into the CNS	[11,78]
	Restoration of blood-brain-barrier properties	[79]
Preserved immunocompetence	Less pronounced immune cell depletion in lymphoid organs	[18]
	Preserved B and T cell responses	[49,80]
	Low depletion of innate immune cells, especially tissue resident cells	[48,50]
Active neuroprotection with regression of disability	Induction of neurotrophin producing lymphocytes	[74]
	Preservation of axonal conductance in MOG_35–55_ EAE	[81]

LAG-3: Lymphocyte-activation gene 3; PD-1: programmed cell death protein 1; TCR: T cell receptor; Th: T helper cell; Treg: regulatory T cell.

#### 2.4.1. What We Learned from Animal Models

Despite certain limitations in translation to human disease, especially in MS, animal models paved the way to a deeper understanding of the pathophysiologic processes. Investigation of alemtuzumab effects however was hampered by the lack of cross-reactivity between human and mouse CD52. The generation of transgenic mice expressing human CD52 (huCD52 mice) addressed this issue. These mice were able to mount normal immune responses. The tissue distribution and expression patterns on immune cells were comparable to those seen in humans. Alemtuzumab treatment recapitulated changes in serum cytokine levels and depletion of peripheral blood lymphocytes observed in humans [18]. B cells returned to baseline levels seven to ten weeks post alemtuzumab, T cells recovered more slowly still not reaching baseline after 25 weeks. As stated above, lymphocyte depletion in lymphoid organs was not as profound as in peripheral blood. Notably, even high dose alemtuzumab was not able to deplete more than 50% of single-positive and double-positive thymocytes, despite high CD52 expression. Whether this accounts for a low penetration of alemtuzumab into lymphoid organs or to inefficient, intraparenchymal, cytolytic processes remains elusive. In agreement with investigations in humans, frequencies of CD4^+^CD25^high^FoxP3^+^ cells were found increased, even though expressing equivalent levels of surface CD52 compared to all CD4^+^ T cells. Presumably, this might be related to homeostatic proliferation, as Tregs respond to self-antigens as has been demonstrated for MS patients [56]. Although recapitulating certain aspects of alemtuzumab treatment in humans, the putative physiologic function of CD52 in humans cannot be fully reproduced in huCD52 mice, affecting the significance and translation of results. However, some relevant aspects, such as alemtuzumab’s impact on secondary lymphoid tissues and CNS pathology can only be extensively investigated in animal models.

T cell transfer experiments with lymphopenic animals have demonstrated that homeostatic proliferation relies on TCR-self peptide mediated stimulation resulting in an oligoclonal population skewed towards self-reactivity [58,82,83]. In these processes, IL-7 has been proposed as one of the main drivers. In contrast to CD8^+^ T cells, CD4^+^ T cells undergo less efficient homeostatic proliferation possibly accounting for the slower repopulation of CD4^+^ T cells after alemtuzumab treatment. An IL-7 dependent down-regulation of MHC II (major histocompatibility complex class) on murine dendritic cells was indicated as underlying mechanism [84]. However, over time, thymic T cell recovery gains more importance as demonstrated by increasing clonal diversity [56]. Thymic T cell generation is normally biased towards the CD4 lineage predominantly arising from higher susceptibility to apoptosis of CD8 lineage thymocytes raising the question whether alemtuzumab influences thymic T cell development affecting the CD4:CD8 ratio [85].

A more recent study in huCD52 mice demonstrated preserved function of residual immune cells after alemtuzumab treatment [80]. Cell proliferation and cytokine production of T cells, T cell receptor repertoire, *in vitro* and *in vivo* innate immune cell responses were unchanged compared to sham-treated mice. *In vitro* and *in vivo* T and B cell responses were restored before cell counts returned to baseline. Overall, this reflects the generally preserved immunocompetence of alemtuzumab treated MS patients.

In other animal studies antibodies against murine CD52 (anti-muCD52) were utilized to investigate treatment effects of depleting anti-CD52 antibodies. A single course of anti-muCD52 provided long-term therapeutic benefits in three animal models of MS reflecting different aspects of disease pathogenesis [81]: Chronic experimental autoimmune encephalomyelitis (EAE) induced by MOG_35–55_ (myelin oligodendrocyte glycoprotein) in C57BL/6 mice primarily driven by peptide-specific CD4^+^ T cells; MOG_1–121_ EAE with T and B cell dependent pathology; and PLP_139–151_ EAE in SJL mice mimicking a relapsing-remitting disease course [86]. The clinical effects were accompanied by reduced frequencies of autoreactive MOG-specific T cells and diminished production of pro-inflammatory cytokines. Histological examination of the CNS revealed less infiltrating lymphocytes, demyelination and axonal loss. Electrophysiological assessment showed preservation of axonal conductance in the spinal cord suggesting that anti-CD52 therapy may help to preserve CNS integrity; whether this is related to active neuroprotective properties or promotion of endogenous repair mechanisms remains elusive. Several previous studies indicate, albeit not with certainty, a pathogenic role for myelin reactive T cells in MS [76]; however their frequencies and/or function have not been investigated in alemtuzumab-treated patients so far.

In MS, autoimmune T cells are thought to migrate into the CNS after peripheral activation. Previously, CD52 has been implicated in transendothelial migration of T cells through human umbilical vein endothelial cell monolayers [11]. However, a recent study evaluating immune cell migration showed no impact of anti-muCD52 treatment *in vitro* (transwell assays) and *in vivo* (intra-cerebroventricular lipopolysaccharide administration; thioglycollate-mediated peritonitis). Whether this applies for MS patients has not been investigated so far, but might have important implications for immune surveillance of the CNS especially in the context of PML [78].

Besides an increased migratory propensity of peripheral activated immune cells, a dysfunction or dysregulation of the blood-brain-barrier substantially contributes to the pathologic CNS immune cell infiltration in MS [87]. In a recent study anti-muCD52 treatment restored epithelial barrier function in a murine model of inflammatory bowel disease mainly attributed to depletion of activated T cells. In this respect, alemtuzumab treatment might lead to recovery of blood-brain-barrier integrity in MS [79].

#### 2.4.2. What We Learned from Clinical Application in MS Therapy

Alemtuzumab has proven superior efficacy to s.c. IFNβ-1a over all key clinical trials in RRMS. *Post-hoc* analyses or investigations based on independent cohorts combined *in vitro* and *ex vivo* experimental data with clinical and MRI results acquiring the current knowledge on the mechanisms of action.

In the early studies alemtuzumab treatment was primarily used in SPMS. Despite significantly reduced ARR (from 0.7 to 0.001; *p* < 0.001) and almost no MRI activity over seven years, patients with SPMS showed sustained accumulation of disability with progressive cerebral atrophy on MRI. RRMS patients showed comparable effects on the surrogate markers of inflammatory activity, and—in contrast to SPMS patients—demonstrated a surprising reduction in disability (mean of 1.2 EDSS points six months after alemtuzumab treatment) [20]. These findings further corroborate the different pathophysiologic concepts of RRMS and SPMS, where inflammatory processes predominantly driven by adaptive immunity are opposed to neurodegenerative processes and prevailing innate immunity compartmentalized behind an intact blood-brain-barrier [88]. Alemtuzumab seems to have only limited impact on the latter pathogenic aspects, confirmed by preserved innate immune responses in animal models and low infection incidence after alemtuzumab. A recent case report of alemtuzumab therapy in an AQP4-antibody positive NMO (neuromyelitis optica) patient demonstrated a progressive and lethal disease course with massive CNS monocytic infiltration further substantiating insufficient suppression of the innate immune system by alemtuzumab [89].

However, the sustained improvement in EDSS—confirmed in subsequent clinical trials—exceeded bias factors such as the regression to the mean phenomenon prompting hypotheses on an active neuroprotective potential of alemtuzumab. A *post hoc* subgroup analysis of the CAMMS223 trial revealed regression of disability in patients even without any evidence of MS disease activity immediately before and during treatment, arguing for potential neuroprotective besides anti-inflammatory effects of alemtuzumab [74]. Supporting this notion, peripheral blood mononuclear cells (PBMCs) isolated from alemtuzumab-treated patients were shown to produce increased concentrations of neurotrophic factors *in vitro* promoting survival of neurons and oligodendrocyte precursors [74]. However, clinical relapses and MRI might not be able to detect subtle inflammatory responses in MS, weakening the line of argumentation. Moreover, neurotrophin-producing cells so far have not been located in the CNS after alemtuzumab-treatment. Neurotrophins produced in the periphery have short half-lives and poor blood-brain-barrier permeability severely limiting their ability to enter the CNS [90]. Active neuroprotection by alemtuzumab is an intriguing concept, the long-lasting reduction of disability is in favor of this hypothesis, but actual substantial evidence is missing.

In contrast, there is clear evidence for the strong anti-inflammatory and immune system reprogramming effects of alemtuzumab. The preserved level of immune competence despite long-lasting immune cell depletion and the qualitative changes in immune components strongly support this notion. Moreover, the rate of lymphocyte reconstitution is unrelated to disease activity, accumulation of disability and secondary autoimmune disease after alemtuzumab [91].

In the first three months following alemtuzumab administration, the CD4^+^ T cell pool is mainly composed of CD4^+^ memory cells, probably due to homeostatic proliferation. As repeatedly demonstrated, cells with a regulatory phenotype CD4^+^CD25^high^CD127^low^ were relatively increased within CD4 subsets for nine months after alemtuzumab treatment [56,72]. Whether this is related to an augmented proliferation in response to self-antigens or a result of relative resistance to alemtuzumab-mediated depletion remains elusive. Tregs found post treatment are mainly CD31 negative arguing against a recent thymic origin. Despite relatively increased Treg frequencies, absolute numbers remain low throughout several years. The low recoverable cell numbers complicate the investigation of their suppressive function early after alemtuzumab. However, as stated above, a recent study has shown restored regulatory capacity of CD4^+^CD25^high^ cells from patients, equivalent to healthy controls three and four years post treatment indicating a long-term rebalancing of immune-tolerance networks [56], at least for MS-related autoimmune responses. Accordingly, the immunoregulatory cytokines TGFβ-1 and IL-10 were found to be significantly increased post treatment. The CD4^+^ T cell pool was skewed to Th2 cells, whereas Th1 and Th17 proportions were reduced with marked decreases in IFN-γ, IL-12, IL-17, IL-21, IL-23 and IL-27 serum levels [75]. Moreover, CD4^+^ T cells showed an upregulation of the inhibitory receptors PD-1 and LAG-3 [56].

Particular T cell clones are expanded in the CNS of MS patients and identical T cell clones unique to each patient are present at distinct anatomical sites suggesting limited numbers of T cell clones contributing to the local immune reactions in MS [92]. A restoration of T cell diversity might therefore have beneficial effects to MS disease activity. In the first months after alemtuzumab treatment, TCR diversity is reduced due to homeostatic proliferation. However, Jones and colleagues reported on a patient providing fast recovery of TCR diversity after six months exceeding pretreatment levels at 12 months [56]. Whether this can be transferred to a larger cohort and relates to a higher treatment efficacy suggesting a possible marker for treatment response has not been investigated so far.

Besides the T cell pool, qualitative changes were also shown for the B cell pool. For at least one year after alemtuzumab treatment, the blood B cell pool is dominated by immature B cell subsets driven by increased serum levels of BAFF. CD27^+^ memory B cells reach only 25% of baseline at 12 months probably accounting to prolonged T cell lymphopenia since T cell help is required for memory B cell development [21].

Thus, the long-term control of disease by alemtuzumab in RRMS patients may involve sustained quantitative and qualitative alterations mainly in T and B cell subsets.

### 2.5. Perspectives and Future Challenges

Alemtuzumab further expands the armamentarium of RRMS therapy comprising now 10 approved agents. Among these alemtuzumab represents a unique therapeutic approach reprogramming the immune system targeting T and B cell compartments with unprecedented durability and long-lasting freedom of disease activity/NEDA (defined as absence of clinical disease and MRI activity). However, with emerging options choosing the adequate agent for the right patient becomes more difficult. Despite no available data, risks of alemtuzumab therapy probably outweigh benefits in patients with mild disease or clinically isolated syndrome, which is reflected by the current EMA (European Medicines Agency) label restricting alemtuzumab use to patients with active RRMS. The German guidelines define active disease as ≥1 relapse under DMT with nine T2-hyperintense lesions or ≥1 Gd-enhancing lesion or ≥2 relapses with EDSS progression and significant MRI disease activity in the past year. Fingolimod and natalizumab represent further highly effective treatment options approved by the EMA for active RRMS. Head-to-head studies comparing efficacy of these agents are still missing. Different comparators (mostly placebo controlled, only fingolimod tested against lower-dose i.m. (intramuscular) IFNβ-1a [93]), varying endpoints and study populations further complicate the comparison of these therapies across clinical studies (Table 5). Therefore, therapy selection is currently primarily guided by other factors including risk stratification (PML risk, cardiac history, family history of autoimmune diseases *etc.*), mode and frequency of administration as well as anticipated adherence to therapy and safety monitoring. Especially for risk stratification the irreversibility of alemtuzumab effects—not seen for other DMTs—has to be considered in patient and therapy selection. The final therapy decision has to be preceded by an in-depth discussion of risks and benefits with the patient.

**Table 5:** Efficacy endpoints of disease-modifying therapies for active RRMS.

Drug/Study	Fingolimod FREEDOMS ^a^ [94,95]	Fingolimod TRANSFORMS ^b^ [93,96]	Natalizumab AFFIRM ^a^ [97,98]	Alemtuzumab CARE-MS I [26,31]	Alemtuzumab CARE-MS II [27,30]
Comparator	Placebo	i.m. IFNβ-1a	Placebo	s.c. IFNβ-1a	s.c. IFNβ-1a
ARR, relative reduction to comparator	54% (*p* < 0.001)	38% (*p* < 0.001)	68% (*p* < 0.001)	55% (*p* < 0.0001)	49% (*p* < 0.0001)
NEDA *vs.* comparator	33% *vs.* 13% (*p* < 0.001)	46% (0.5 mg), 38% (1.25 mg) *vs.* 34% (*p* < 0.001, *p* = 0.18)	37% *vs.* 7% (*p* < 0.0001)	Y1: 50% *vs.* 38% (*p* = 0.0053)	Y1: 44% *vs.* 27% (*p* = 0.0001)
				Y2: 68% *vs.* 47% (*p* < 0.0001)	Y2: 58% *vs.* 32% (*p* < 0.0001)
				Y3: 62%	Y3: 53%
				Y4: 60%	Y4: 55%

^**a**^: study data over 24 months; ^**b**^: study data over 12 months; ARR: annualized relapse rate; IFNβ-1a: interferon β-1a; i.m.: intramuscular; NEDA: no evidence of disease activity (no Gd + T1 lesions, new/enlarging T2 lesions, no confirmed relapses or 3-month (fingolimod, natalizumab)/6-month (alemtuzumab) sustained disability progression); s.c.: subcutaneous; Y: year.

New markers for treatment response and risk stratification guiding patient selection are urgently needed. Earlier evidence of IL-21 (or a combination of IL-21 and IL-7) as a potential biomarker has not been substantiated in larger prospective cohorts, further complicated by the inability of currently available IL-21 ELISA kits to predict autoimmunity after alemtuzumab [99]. Reliable markers for treatment response are also missing. Previous reports of differential lymphocyte reconstitution as a potential biomarker for relapse risk post-alemtuzumab treatment were not substantiated in other cohorts [91,100]. This extends to the important and open clinical issue of re-treatment. The current labeling information contains no recommendations when and how to retreat patients with alemtuzumab after the initial two courses. In CARE-MS I and II trials, patients were offered additional courses when a relapse or ≥2 new MRI lesions occurred; whether repeated courses will affect safety remains uncertain. In a recent seven-year follow-up study of 87 patients, relapses prompted retreatment with one additional course in 36%, two in 8% and three in 1%; however the number of treatment courses was not associated with incidence of secondary autoimmune disease [16].

As previously suggested another option would be to use alemtuzumab as induction therapy based on the long-lasting qualitative changes of the immune-system and followed by established DMTs. Although, this concept might have far-reaching implications it will require large cohort studies with long-term follow-up and complex study designs. An initial safety exploration of DMTs after alemtuzumab revealed no additional risk [101].

So far, the follow-up studies have not revealed new safety signals, nevertheless uncertainty of unknown AEs remains, especially for long-term risks such as malignancy, issuing challenges in continuous patient surveillance and education.

Besides urgently needed measures for individual risk stratification, further strategies to reduce the risk of secondary autoimmune disease are under investigation or have been suggested. Currently keratinocyte growth factor treatment in combination to alemtuzumab therapy is tested in RRMS patients to facilitate thymic T cell recovery rather than homeostatic proliferation (ClinicalTrials.gov: NCT01712945). A combination with rituximab has been suggested to prevent B cell mediated secondary autoimmune disease.

Moreover, new compounds targeting CD52 might provide an improved safety profile. The compound GZ402668 was generated using germline sequences and utilizing *in silico* prediction algorithms to achieve reduced immunogenicity. The *in vitro* and *in vivo* (in huCD52 mice) lytic activity was similar to alemtuzumab, whereas the release of the proinflammatory cytokines IFN-γ and TNF-α by human blood cells was reduced indicating a potentially lower risk for IARs. GZ402668 is currently being tested in a Phase I clinical trial in MS patients (ClinicalTrials.gov: NCT02282826) [102]. Immunogenicity might also induce antiglobulin responses to alemtuzumab, especially after repeated infusions in long-term treatment, potentially affecting—albeit not observed so far—treatment efficacy. Pretreatment with a high dose non-cell-binding variant of alemtuzumab has been shown to reduce antiglobulin responses; whether patients might benefit from this approach remains elusive [103].

Given the high efficacy of alemtuzumab, the indications of alemtuzumab might expand to other B or T cell driven autoimmune diseases with currently limited therapeutic possibilities such as idiopathic inflammatory myopathies, and warrant further studies [104,105].

## 3. Conclusions

Alemtuzumab is a promising new agent in the armamentarium of MS therapy. The long durability with sustained reprogramming of the immune system targeting T and B cells provides a unique treatment approach for MS with potential impact on the causes underlying the disease. However, the significant risks have to prompt extensive monitoring and patient education, and demand reliable markers for risk stratification and treatment response improving patient selection and therapy guidance. The potential irreversibility of alemtuzumab’s effects, with no means of antagonisation and the window of therapeutic opportunity in early MS disease phases further stress this significant unmet need. Only the understanding of mechanisms of action (MOA) will allow for the best possible risk-benefit ratio for the individual patient. Further investigation into the MOA with a high-resolution, single-center study (ALAIN01: Alemtuzumab in Autoimmune Inflammatory Neurodegeneration: Mechanisms of Action and Neuroprotective Potential; ClinicalTrials.gov: NCT02419378) is currently being pursued.
